# Selecting biologically informative genes in co-expression networks with a centrality score

**DOI:** 10.1186/1745-6150-9-12

**Published:** 2014-06-19

**Authors:** Francisco J Azuaje

**Affiliations:** 1 NorLux Neuro-Oncology Laboratory, Centre de Recherche Public de la Santé (CRP-Santé), Luxembourg, Luxembourg

**Keywords:** Network hubs, Weighted networks, Gene co-expression networks, Centrality scores, Zebrafish, Heart regeneration, Cancer, Microarrays, RNA-Seq

## Abstract

**Background:**

Measures of node centrality in biological networks are useful to detect genes with critical functional roles. In gene co-expression networks, highly connected genes (i.e., candidate hubs) have been associated with key disease-related pathways. Although different approaches to estimating gene centrality are available, their potential biological relevance in gene co-expression networks deserves further investigation. Moreover, standard measures of gene centrality focus on binary interaction networks, which may not always be suitable in the context of co-expression networks. Here, I also investigate a method that identifies potential biologically meaningful genes based on a weighted connectivity score and indicators of statistical relevance.

**Results:**

The method enables a characterization of the strength and diversity of co-expression associations in the network. It outperformed standard centrality measures by highlighting more biologically informative genes in different gene co-expression networks and biological research domains. As part of the illustration of the gene selection potential of this approach, I present an application case in zebrafish heart regeneration. The proposed technique predicted genes that are significantly implicated in cellular processes required for tissue regeneration after injury.

**Conclusions:**

A method for selecting biologically informative genes from gene co-expression networks is provided, together with free open software.

**Reviewers:**

This article was reviewed by Anthony Almudevar, Maciej M Kańduła (nominated by David P Kreil) and Christine Wells.

## Background

The analysis of gene co-expression networks has become an important approach to enabling fundamental and translational biomedical research. Such transcriptional association networks have allowed the generation of novel hypotheses about potential functional roles of genes or about their involvement in phenotype-specific cellular processes [1-5]. In these networks, genes and their co-expression relationships are graphically represented as nodes and edges respectively. Network edges are typically estimated with measures of expression correlation, such as the Pearson’s correlation coefficient, which have shown to be powerful predictors of biologically interesting relationships [6]. The resulting networks can be analyzed using different methods based on statistical and graph theory concepts, which link network topological features to biologically informative properties.

Among these techniques, different measures of *node centrality* have been proposed to identify functionally-critical network components. The *degree* of a node, defined as the number connections associated with a gene, is one such measure of centrality. The Methods section introduces different traditional measures of node centrality. In bioinformatics, centrality measures have been applied to describe the global structure of networks [4,5]. The potential utility of centrality indicators in gene co-expression networks have been reported in various application domains, such as cardiovascular and cancer research [7-10]. For instance, genes exhibiting high degree or high betweenness-centrality scores have been proposed as candidate targets in different human and animal models [8-10].

Despite these promising advances, deeper investigations of node centrality applications in gene co-expression networks are still needed. A key issue is that standard measures of gene centrality are typically applied to co-expression networks that are defined with binary interactions. In this traditional scenario, a network edge indicates that the co-expression between a pair of genes is observed above a pre-defined correlation threshold. The definition of correlation thresholds is a non-trivial task for which there is no standard approach [11]. Estimators of gene connectivity have mainly been applied to summarize and compare the global structure of co-expression networks. Moreover, traditional approaches are limited by the notion of defining candidates hubs by either counting the number of edges assigned to a node or by estimating the total intensity of the connections without providing an indicator of statistical significance (*P* value) for each candidate hub. Therefore, although gene centrality approaches have shown to be useful to extract novel knowledge, important insights into the diversity of co-expression values and their statistical relevance may be missed.

Key biological premises that motivate the analysis of gene co-expression networks on the basis of centrality measures are: a. Highly co-expressed genes are more likely to be co-regulated, and b. Those genes that display prominent connectivity patterns tend to play biologically influential or regulatory roles in disease-related processes. Here, I test these notions through various indicators of node centrality in different gene co-expression networks, which were generated from three research application areas and two expression measurement platforms. Furthermore, researchers traditionally detect candidate network hubs by counting the number of edges associated with a node. In the context of gene correlation networks, a connection is typically defined if the correlation between a pair of genes is above a predefined cut-off value. Also there is a need to offer an automated way to quantify (and rank) the resulting candidate hubs according to the statistical significance of their observed connectivity.

Here, I report a method that identifies biologically informative genes, i.e., candidate hubs, based on their co-expression values and corresponding indicators of statistical relevance. This strategy does not require the selection of co-expression thresholds, and enables a characterization of the strength and diversity of co-expression relationships in the network. I show that it can outperform and complement standard centrality measures. I illustrate gains in terms of the prediction of biologically meaningful genes. This method can identify and rank gene sets with high statistical confidence and with larger enrichments of cellular processes. Moreover, a deeper look into one of the networks, a case study on zebrafish heart regeneration, allowed the identification of genes and pathways with known and potentially novel driving roles in tissue regeneration after injury, *in vivo*. The proposed method and resulting free software will help researchers to focus their attention on genes that may be biologically informative in their gene co-expression network investigations.

## Methods

### Weighted node connectivity

One way to account for co-expression intensity consists of assigning weights to each edge in the network. In the case of gene co-expression networks, an edge weight, between genes *i* and *j*, can represent the value of the expression correlation coefficient between genes *i* and *j*. I studied a weighted version of degree, which has previously been applied to the analysis of traffic control and social networks [12,13]. In bioinformatics it has been applied to compare the global structure of networks [1,5,14]. Previous investigations, however, have mainly addressed gene selection based on the analysis of the observed connectivity values only, without statistical significance inference at the level of individual genes. The method reported here goes beyond this by implementing a non-parametric statistical assessment procedure.

The weighted node connectivity, *WNC*, score can be specified as:

(1) $$\mathrm{WN}C_{i}=\;\sum_{j}^{N}w_{i,j}$$

where node *i* is connected to node *j*, and *w*_*i,j*_ reflects the strength of the connection of node *i* with node *j* (input information provided by the user). The algorithm is not constrained by the correlation measurement used to generate the input network. In this paper, *w*_*i,j*_ is computed as the absolute value of the Pearson correlation coefficient between genes *i* and *j*.

Previous research, including those cited above, shows that *WNC* is a reliable indicator of connectivity based on co-expression information. More importantly, these investigations have shown that the score is useful to explore the potential biological significance of genes. For instance, they showed that the score is a good indicator of biological relevance when searching for possibly interesting groups of interconnected genes, i.e., network modules [1,14].

For each gene, I calculated the *WNC*_i_ score and support each value with an indicator of its statistical significance. This is needed for reducing noise and possible bias toward many connections with weak *w*_*i,j*_ values, as well as for ranking the selected genes. Thus, I estimated *P* values for each *WNC*_i_ score. This was done by implementing a permutation-based test that compares the observed *WNC*_*i*_ with an (empirical null) distribution of random *WNC*_*i*_*,*_*random*_ values. The latter was estimated by randomly swapping the edges in the network, while preserving the distributions of observed edges and weights in the network. Given two randomly selected edges: e(*x*,*y*) and e(*v*,*z*) with corresponding weight values *w*_*x,y*_ and *w*_*v,z*_, where: *x,y,v,z* represent network nodes and *x ≠ y ≠ v ≠ z*, the edge weights *w*_*x,y*_ and *w*_*v,z*_ are assigned to e(*v,z*) and e(*x,y*) respectively. Thus, the resulting null distribution is both random and feasible. This edge randomization procedure has been applied to estimate null distributions in different network research applications [15-17]. A *P* value is obtained by calculating the proportion of *WNC*_*i*_ ≥ *WNC*_*i*_*,*_*random*_, where the *WNC*_*i*_*,*_*random*_ values were obtained from 100E + 3 permuted samples. This permutation procedure is suitable to generate a null model because the resulting networks are random and comparable with the observed (original) network. In particular, this procedure guarantees the preservation of fundamental properties of the original network: size, degree distribution and weight distribution [18]. Furthermore, each *P* value is adjusted with a Bonferroni correction to account for multiple testing. This correction provides sufficiently conservative estimates of “significance” among the many statistically detectable scores. Those *WNC*_*i*_ with (adjusted) *P* < 0.05 are here reported as relevant and considered for further analysis.

Ranking and selection of genes should be based on their *P* values. This is because, in comparison with *WNC* scores, *P* values provide a more reliable estimator of gene connectivity. Indeed, it is possible to obtain genes with relatively high *WNC* scores that are detected as statistically spurious after *P* value calculation. This is reflected in the observation that *WNC* scores and their corresponding *P* values are not perfectly linearly correlated (Additional files 1, 2 and 3). In this study, Pearson correlations between *WNC* scores and *P* values range from -0.53 to -0.85 in different networks. Moreover, *P* values represent more interpretable values to bioinformaticians and experimental biologists alike, e.g., values between 0 and 1 with well-defined statistical meaning.

Software and published work based on random permutation methods, including network-based research, typically define between 1 K and 10 K random samples for networks of similar or larger size [17,19]. In the case of my algorithm, when tested on different datasets, a number of permuted samples above 10 K gives highly consistent estimations of *P* values. Indeed, there are no detectable differences in the datasets investigated here when generating more than 50 K permutated samples. Thus, although there is no guarantee that my test can estimate the true exact *P* values, these observations at least indicate that the selected number of permutated samples is sufficiently large. Also it is important to highlight that the accompanying software allows users to define this parameter according to their needs and computing resources.

### Other indicators of gene centrality

I also investigated 4 standard indicators of node centrality, which have previously been applied to diverse biological research applications. These scores are applied to networks in which edges are defined as binary interactions, i.e., an edge, *a*_ij,_ connecting genes *i* and *j* is represented with a value of 1 if the expression correlation between genes *i* and *j* is above a predefined value, otherwise *a*_ij_ is equal to 0.

a. The *degree*, *d*_i_, of gene *i* in a network of *N* genes, represents the number of nodes connected to gene *i*[20]. Genes with large degrees are commonly referred to as hubs. This indicator can be defined as:

(2) $$d_{i}=\;\sum_{j}^{N}a_{\mathrm{ij}}$$

Where *a*_ij_ is 1 if gene *i* is linked to gene *j*, and 0 otherwise.

b. The *closeness centrality*, *c*_i_, encodes the capacity of node *i* to interact with all the other nodes in the network, including those that are not directly connected to node *i*[21]. This measure is calculated as the inverse sum of the shortest distances from *i* to all the other nodes in the network:

(3) $$c_{i}=\;\frac{1}{\sum_{j}^{N}h\left(i,j\right)}$$

Where *h*(*i*,*j*) represents the shortest distance between genes *i* and *j*.

Equation (3) will only return values for nodes that are connected to other nodes. Also the closeness centrality value of a node is calculated in relation to the connected graph in which the node is located. This is a key limitation of this approach, which also implies that nodes located in small sub-networks (separated from the largest connected sub-network) may report relatively high closeness centrality values. In this investigation, all unweighted networks analyzed with this score consisted of a large connected graph containing the vast majority of the nodes, and all of the nodes had at least 1 edge and reported closeness centrality values between 0 and 1.

c. The *betweenness centrality*, *b*_i_, encapsulates the property of node *i* as a bridging node in the network, i.e., a measurement of the number of shortest paths connecting any two nodes, *j* and *k*, which pass through node *i*[22]. Nodes with large *b*_i_ values are often called “high traffic” nodes.

(4) $$b_{i}=\;\sum_{i\neq j\neq k}\frac{n_{j,k}\left(i\right)}{n_{j,k}}$$

Where *n*_*j,k*_ is the total number of shortest paths connecting nodes *j* and *k*, and *n*_*j,k*_(*i*) is the number of shortest paths that pass through node *i*.

d. The *clustering coefficient*, *u*_*i*_, indicates the edge density in the neighborhood of node *i*. This is calculated as the proportion of edges that exist between the nodes contained in its neighborhood, divided by the total number of edges that can potentially be found between these nodes [23]:

(5) $$u_{i}=\;\frac{m_{i}\left(j,k\right)}{m_{i}}$$

Where *m*_*i*_(*j,k*) is the number of edges connecting all nodes *j* and *k* in the neighborhood of node *i*, and *m*_i_ is the total number of edges that could be seen if all the nodes in the neighborhood of *i* were fully connected.

### Implementation of scores

The *WNC* score was implemented in Java. Source code and an executable program, WiPer, are freely available (see Software Availability). The centrality measures specified in formula (2) to (5) were computed with the Network Analyzer plugin [24] of Cytoscape [25].

### Datasets

Gene expression datasets from published studies on: glioblastoma treatment (GBM), kidney versus liver tissue comparisons (KL) and heart regeneration after injury in zebrafish (ZF) were analyzed [26-28] (see Table 1).

**Table 1:** The gene expression datasets investigated

**Dataset**	**Biological problem**	**Tissue origin**	**No. genes**	**No. samples**	**Platform**	**Source**
GBM	Glioblastoma resistance to treatment	Brain	198	10	Microarrays	[26]
KL	Comparison of tissue types	Kidney and liver	211	14	RNA-Seq	[27]
ZF	Zebrafish heart regeneration after amputation	Heart	221	9	Microarrays	[28]

To minimize noise and facilitate interpretation of results, genes showing significant expression changes across samples were selected for network generation. In the GBM dataset, I focused on 198 genes displaying │log_2_Fold-Change │ ≥ 1 between treatment and control samples. In the KL dataset, 211 genes showing high differential expression between kidney and liver samples were selected (those with adjusted *P* = 0). In the ZF dataset, I selected 221 genes, which showed statistically detectable differential expression between 1- and 7-day post-injury in relation to uninjured (control) samples (adjusted *P* < 0.05). “Adjusted *P*” refers to *P* values obtained after correcting for multiple testing. In the GBM dataset, log_2_Fold-Change was used to select genes, instead of *P* values, because the latter detected relatively low numbers of differentially expressed genes. In the other datasets, the chosen *P* values allowed the selection of gene sets consisting of hundreds of genes instead of thousands. In all datasets, the latter was the main selection requirement. Microarray data analyses were implemented with the limma package (MEV platform, v4.9.0) [29,30]. DESeq tools (version 1.10.1) were applied to the RNA-Seq dataset under the R platform (v. 2.15.3) [31].

### Generation of gene co-expression networks

For all pairs of genes in the datasets, the Pearson correlation coefficient, ρ, was calculated to define the strength of the gene-gene connections in the network. In the analyses of *WNC* scores, I considered all the obtained ρ values to generate the networks (weighted networks). As both negative and positive correlations may be biologically interesting, network edges were represented with absolute ρ values, │ρ_i,j_ │. For the analyses of standard centrality measures (unweighted networks), I concentrated on gene co-expression values sufficiently high to reduce the possibility of including spurious associations and to focus on the most likely relevant co-expression patterns. Here I report results in which a network edge was established, between nodes *i* and *j*, if │ρ_i,j_ │ ≥ 0.95-percentile, i.e., those │ρ_i,j_│ values falling above the 95th percentile of the observed data. Although this procedure is expected to reduce the number of edges in comparison to the weighted network, the total number of genes is not substantially affected. The characteristics of the resulting networks are summarized in Table 2. Gene co-expression values were computed with the MINE application [32], and networks were visualized with Cytoscape [25]. Note that the proposed *WNC* score does not require the application of specific gene selection criterion or correlation measures to generate the networks. Here, to facilitate comparisons, the same gene selection criterion (inputs to network generation) and correlation measure were applied to generate the weighted and unweighted networks in each dataset.

**Table 2:** The gene co-expression networks investigated

**Dataset**	**Network type**	**No. nodes**	**No. edges**
GBM	Weighted	198	19530
KL	Weighted	211	22155
ZF	Weighted	221	24310
GBM	Unweighted	143	976
KL	Unweighted	180	1109
ZF	Unweighted	208	1216

To illustrate the application of the algorithm, other co-expression cut-off criteria for network generation are feasible as well. Here, changes in these thresholds naturally will have the effect of generating different network sizes and, therefore, different numbers of putative hubs. Nevertheless, for conservative threshold selections, e.g., cut-offs above the 75th percentile, I have found that hubs detected as highly significant will consistently appear as top-ranked genes in different network generation settings.

### Exploration of potential biological relevance

Here, a group of genes are considered as biologically informative if at least there are statistically detectable associations between the genes and biological processes relevant to the phenotype under investigation. The potential biological relevance of the predicted genes can be estimated using an independent information resource that encodes associations between gene sets and biological processes.

The potential biological relevance of the genes identified as significant with the centrality scores was first estimated through an enrichment analysis of Gene Ontology (GO) terms. Using Fisher’s exact test and multiple-test corrections, this procedure aimed to detect biological processes that are statistically associated with the genes selected. This was done for functional terms in the biological process hierarchy of GO, from levels 3 to 9, and considered as significant when *P* < 0.05. Analyses were implemented with Babelomics (v. 4.3) [33]. An independent estimation of GO biological process enrichment (at all levels) was completed with DAVID (v. 6.7), with those terms reporting FDR < 0.05 considered as statistically significant [34].

This analysis was extended in a deeper investigation of the application case in zebrafish heart regeneration. Based on the integration of independent datasets, I computationally predicted biological processes that are statistically associated with the top-ranked genes (*P* < 0.05). These analyses were performed with the IMP (Integrative Multi-species Prediction) system [35], and associations with biological processes were deemed significant at *P* < 0.05. The IMP model is based on the integration of multiple sources of biological information, including protein-protein interactions and gene expression profiles [35].

## Results

### *WNC* scores are statistically discernible

**Figure 1:**
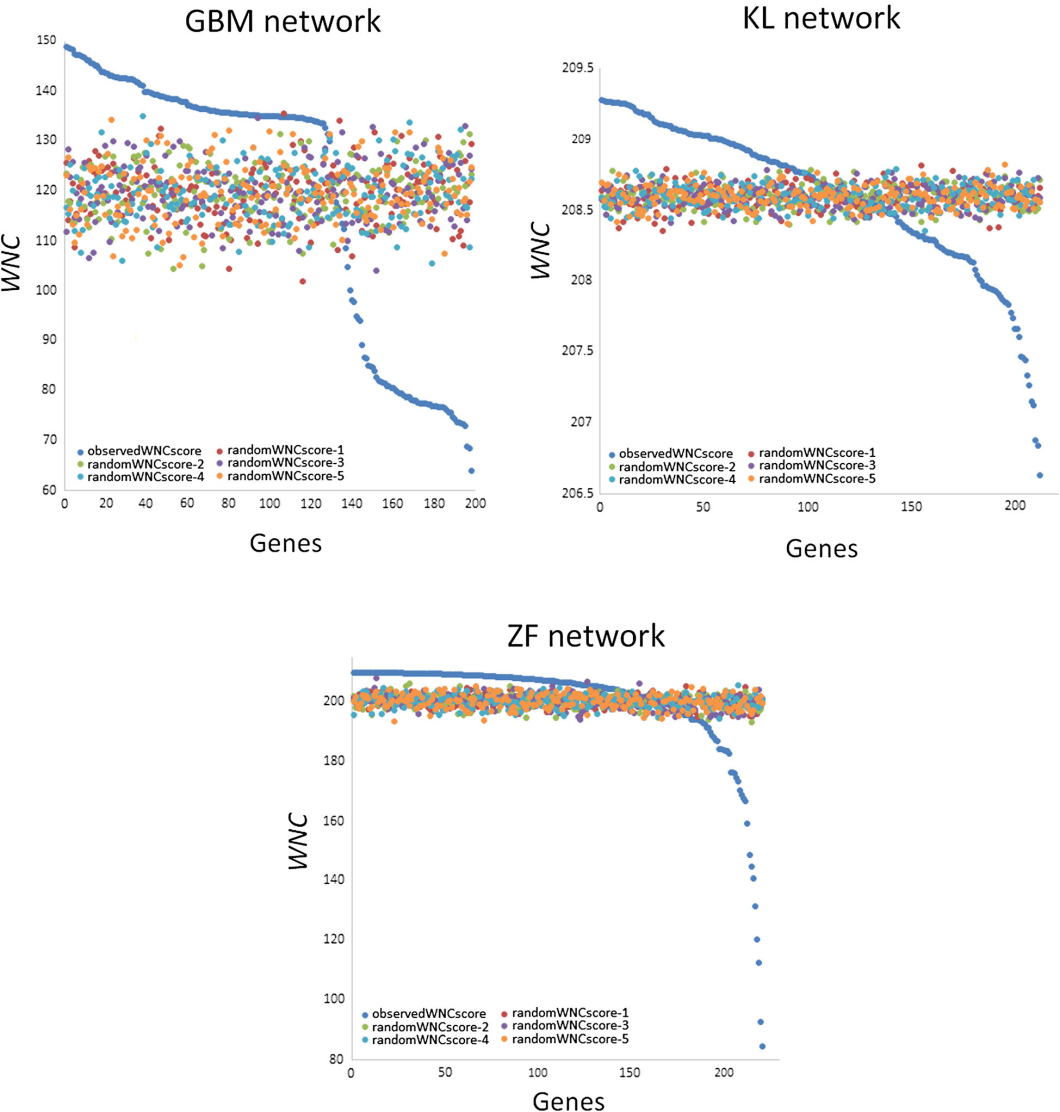
**Distribution of *WNC *scores in real and permuted co-expression networks.***WNC* values from the real and permuted networks are color-coded. To give a sense of the distribution of random samples, the results from 5 permuted networks are shown.

First, I examined whether *WNC* scores observed in real co-expression networks can be distinguished from those obtained from randomly permuted versions of these networks. This was done by comparing the distributions of observed vs. permuted *WNC* values for all the genes in the three weighted co-expression networks (Methods). I found that real networks tend to display *WNC* scores that are higher than those obtained from permuted networks. Figure 1 portrays the distribution of scores measured in the real and permuted networks. To facilitate visualization, results from only 5 permuted networks (per dataset) are shown.

These results indicate that it is possible to distinguish between the *WNC* value distributions from real and random networks. The distributions from real networks contain more extreme lower and upper values, which are also widely separated. In the permuted networks, the *WNC* value distributions tend to be much flatter. This was particularly clear in the ZF and KL datasets. In the KL dataset, for example, the random *WNC* scores ranged from 208 to 209. Taken together, this shows that: a. real gene co-expression networks consist of larger gene sets with extreme *WNC* scores than random networks, and b. *WNC* scores together with a random sampling procedure could be useful to filter spurious associations.

### Identification of phenotype-related genes based on *WNC* analysis

I developed an algorithm for computing *WNC* scores and their corresponding *P* values based on a random permutation test (Methods). An executable program is freely available (Software Availability).

**Figure 2:**
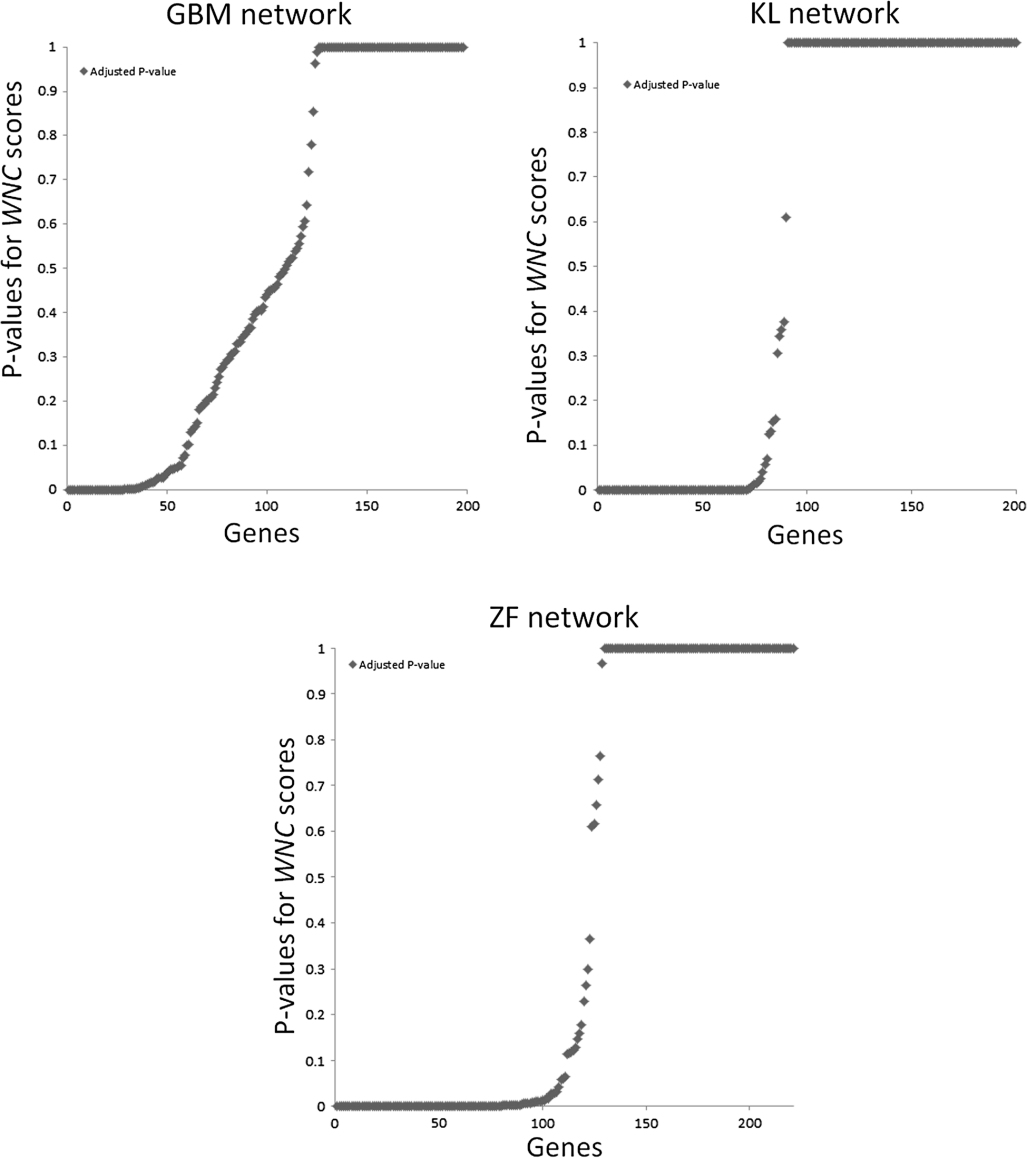
**Identification of statistically significant genes based on *WNC *scores.** For each network, the adjusted *P* values computed for all the *WNC* scores is displayed. Genes with low *P* values represent potential biologically relevant genes and can be used for further analysis.

Figure 2 displays the distribution of adjusted *P* values estimated in the three networks. In each case, relatively small subsets of genes were detected as statistically significant, as expected (Additional files 1, 2 and 3). The GBM network reported the smallest set of significant *WNC* values (55 genes), while the ZF network contained the largest set (99 genes). In the KL network, 79 genes showed highly significant *WNC* scores.

**Figure 3:**
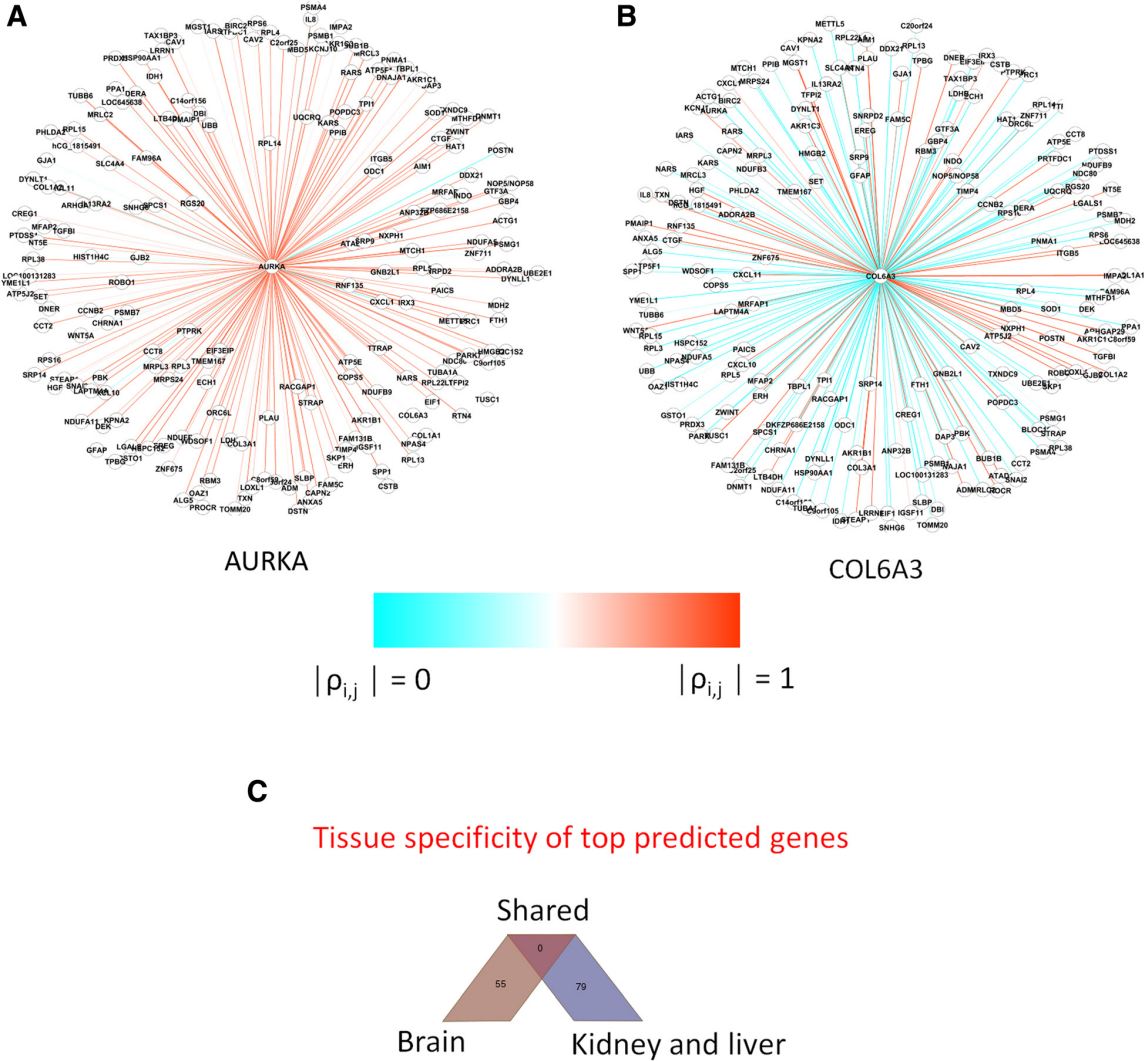
**Examples of top genes with highly significant *WNC *scores in the GBM network.** Genes detected with *WNC* scores are shown as central nodes with their respective co-expression relationships. Panel **A** shows an example of gene with significant *WNC* score (AURKA, *P* = 0). Example of a gene with a statistically spurious *WNC* (COL6A3, *P* =1) is shown in **B**. Edges are color-coded to reflect the intensity of the gene co-expression values. **C**: top predicted genes from GBM and KL networks do not overlap, which suggest tissue specificity of the observed associations.

The main hypothesis is that genes exhibiting statistically detectable *WNC* scores, i.e., those with low *P* values, are informative to the phenotypes investigated. Such genes are candidates for further studies, including analysis of their involvement in diverse cellular processes. Figure 3 shows examples of genes identified as highly significant. The difference between statistically strong and weak *WNC* values is also illustrated. Genes detected as highly significant tend to have many strong connections (Figure 3A). This is illustrated with the gene AURKA (aurora kinase A), which includes many strong (red edges) connections reporting a *WNC* with *P* = 0. Moreover, as desired, nodes displaying a relatively large number of weak connections are not guaranteed to generate statistically detectable *WNC* scores (Figure 3B). The latter is exemplified with COL6A3 (Collagen, Type VI, Alpha 3), which displays a large number of relatively weak connections (cyan edges) and reported a statistical significance of *P* = 1. AURKA is a kinase known to be implicated in the regulation of cell cycle progression and has been associated with different tumor types and treatment responses [36,37]. COL6A3 is a collagen protein that has been linked to different inherited muscular disorders [38]. The AURKA network includes 109 associations with absolute Pearson correlations above 0.80, including 36 correlations above 0.90. They include genes involved in the regulation of apoptosis: BLOC1S2, CSTB, LGALS1, PRDX3 and RTN4, and in microtubule cytoskeleton organization: ZWINT and BLOC1S2 (David tool). This set of highly correlated genes also includes IDH1 (Isocitrate Dehydrogenase 1), which has been proposed as a potential therapeutic target in gliomas [39]. In contrast, the COL6A3 network includes 120 associations with absolute Pearson correlations below 0.30. This low-correlated set includes genes linked to a variety of cellular processes ranging from protein translation (such as KARS and COPS5), RNA processing (such as RBM3 and RPL14) to metabolism (such as DERA and ATP5F1).

Next, I found evidence of the capacity of the algorithm to detect biologically informative candidates. In the GBM network, genes with known impact in key GBM-related processes, such as metabolic perturbations and signaling of cell division, were found in the set of top-ranked predictions (Dataset S1). A prominent example is AURKA (*WNC* with adjusted *P* = 0), which has been recently linked to tumorigenicity and cell self-renewal in GBM [40]. Moreover, 8 of the top-ranked candidate genes in the GBM network were significantly associated with cellular metabolism (generation of precursor metabolites and energy, adjusted *P* = 8.9E-03).

The sets of highly significant genes identified in the human networks did not overlap. None of the 55 genes found as potentially interesting candidates in the GBM network were found in the set of 79 top-ranked genes in the KL network (Figure 3C, Additional files 1 and 2). This indicates that these predictions are particular to these two organ-specific networks. This may offer further indication of the potential of the method to pinpoint biologically informative, highly central genes. A deeper look into the potential predictive value of this technique in the context of the ZF network is presented in the application case section.

### Comparison with standard gene centrality methods

The standard node centrality measures were applied to the three datasets (Methods), and comparisons with the *WNC*-based results were implemented. In the case of the standard measures, the genes were ranked according to their score values, and those genes with the highest values were selected for the comparisons. Unlike the *WNC* score, these indicators do not offer a straightforward statistical criterion to select the most relevant genes. Here, for each network and centrality score, I focused on genes with centrality scores above the 0.95 percentile of the observed distribution in each network. This has two advantages: a. the definition of “high score” is network-specific, and b. this is less biased than the application of an arbitrary score cut-off for all datasets. The sizes of the gene sets obtained for each method and dataset are shown in Table 3.

**Table 3:** Top-ranked gene sets included in the comparison of gene centrality scores

**Method**	**GBM network**	**KL network**	**ZF network**
*WNC*	55	79	108
Closeness centrality	10	17	12
Betweenness centrality	10	17	18
Clustering coefficient	14	22	23
Degree	12	18	15

Number of genes are indicated for each method and dataset investigated.

The different methods independently identified partially overlapping gene sets. There was no gene set shared by all the 5 methods. As expected, the *WNC* and (unweighted) degree scores tended to detect genes in common in all the compared networks. However, the degree score did not consistently show the largest commonality of results with *WNC*. Betweenness centrality, for example, shared common genes with *WNC* in the KL (10 genes) and ZF networks (2 genes), which is comparable to the (unweighted) degree-derived results. In the GBM network, the *WNC*, closeness and betweenness centrality scores detected AURKA. In this network, the largest overlapping gene set was shared by the *WNC* and degree methods (5 genes). In the KL network, *WNC*, closeness centrality and degree identified C19orf77 (chromosome 19 open reading frame 77) as a highly central node. Betweenness centrality was followed by the clustering coefficient as the method exhibiting the largest set of shared genes with *WNC* (3 genes). In the ZF network, the degree score showed the largest overlap with *WNC* (13 genes), followed by betweenness centrality (5 genes). These three methods together only reported 2 (unannotated) genes in common as potentially significant.

**Figure 4:**
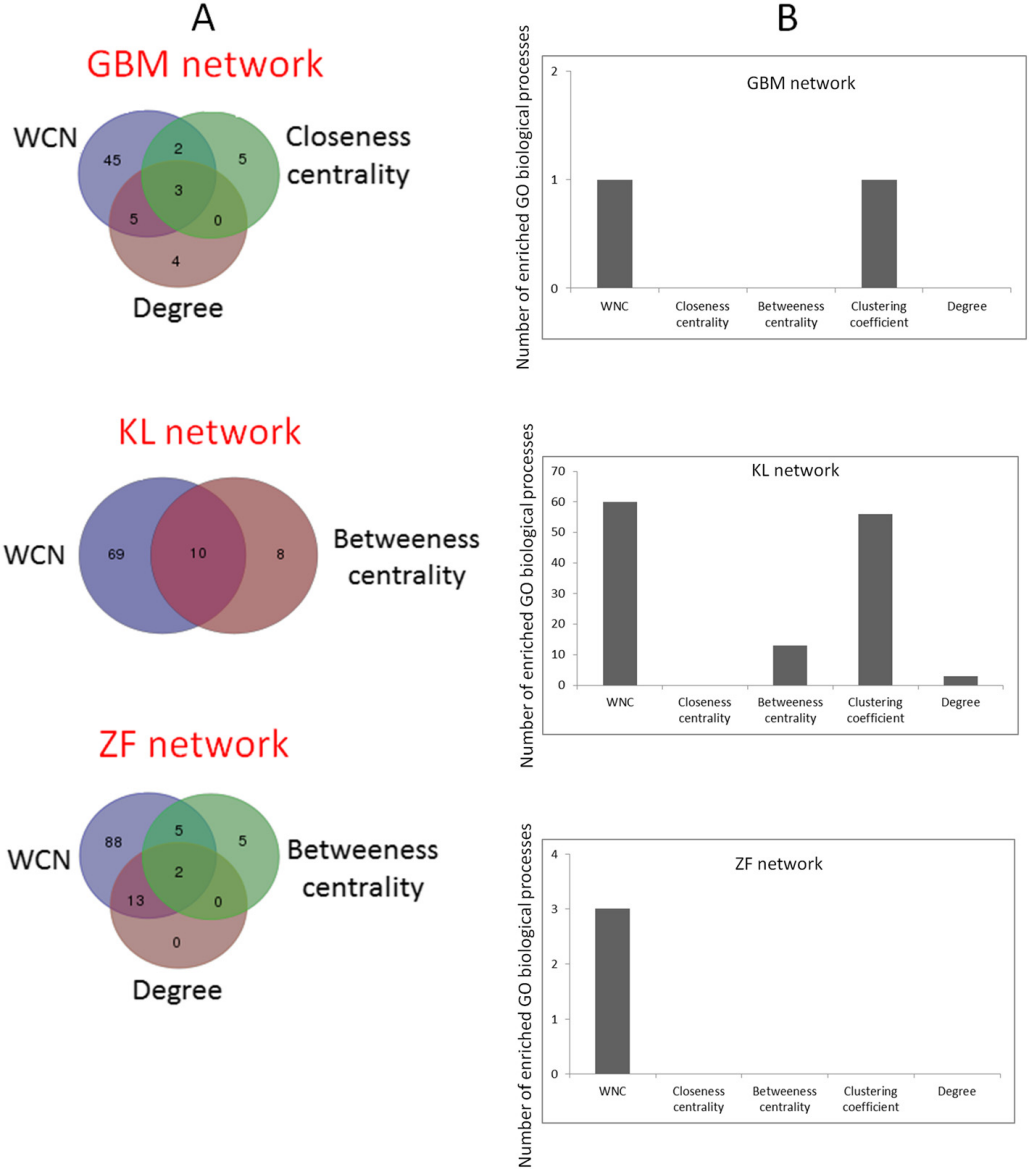
**Comparison of gene sets identified by *WNC *and standard centrality scores. A**. Venn diagrams depicting overlaps between the gene sets identified by the different methods. Only the largest overlaps with *WNC* are shown to facilitate visualization. **B**. Statistically significant GO enrichments found in the predicted gene sets.

Thus, these results suggest that the *WNC* score can select gene sets that standard methods may not identify. At the same time, *WNC* can detect potentially relevant genes that are also independently detected by other methods. Figure 4A summarizes these comparisons. For clarity, only the largest overlaps with the *WNC* method are graphically depicted.

The enrichment of biological processes (Methods) of the genes selected by the different methods was compared. Figure 4B summarizes the results from the 3 datasets. In the GBM network, only *WNC* and the clustering coefficient predicted genes with significant functional enrichments. *WNC* detected a gene set strongly implicated in metabolism (generation of precursor metabolites and energy, *P* = 0.009), and the clustering coefficient offered an enrichment in signal transduction (phosphatidylinositol-mediated signaling, *P* = 4.4E-2). *WNC*, betweenness centrality and the clustering coefficient achieved statistically detectable enrichments in the KL network, with *WNC* providing the largest number (60 processes). In the ZF network, only the *WNC*-based method reported statistically significant enrichments: regulation of immune system process (*P* = 2.4E-2), humoral immune response (*P* = 1.3E-2) and protein processing (*P* = 2.9E-2). Although this comparison does not provide conclusive evidence of the predictive power of this technique in these datasets, it offers further evidence of its hypothesis generation potential.

To further illustrate the importance of statistical assessment in the *WNC*-based technique, a set of top genes ranked exclusively on the basis of their *WNC* values was selected from each network (Additional files 1, 2 and 3). Note that this method for selecting candidate nodes is equivalent to that available in the Weighted Correlation Network Analysis (WGCNA) method [41]. To select these genes, a cut-off number of top genes was defined. To make this comparable to the non-*WNC*-based methods, this number was equal to the maximum number of genes retrieved from the standard methods in the GBM, KL and ZF networks (Table 3). Statistically significant associations with GO biological processes (*P* < 0.05) were not detected in any of these settings.

Although the selection of gene sets from the traditional methods is viable in a typical network analysis, it is necessary to caution that a fairer comparison of performance with the proposed method should be based on size-matched datasets.

Therefore, to investigate the possibility that the perceived advantages of the *WNC*-based predictions over standard techniques is explained by the relatively larger gene sets detected by the former method, an independent GO enrichment analysis that accounts for this difference was implemented. The statistical enrichment of GO biological processes of top-ranked genes from each technique was estimated (DAVID tool, statistical significance at *P* < 0.05), in which the numbers of genes analyzed were equal to those detected by the *WNC* score: 55 genes (GBM network), 79 genes (KL network) and 108 genes (ZF network). In the GBM network, only the clustering coefficient predicted genes significantly enriched in biological processes (3 in total), which is consistent with the results previously obtained. In the KL network, the betweenness centrality score preserved its capacity to detect significant enrichments (17 in total). This time the genes identified by the clustering coefficient did not report significant associations. The degree-based method improved its predictive potential (14 significant enrichments), which is still below the number of statistically detectable associations that the *WNC* score reported. In the case of the ZF network, none of the standard methods reported significant enrichments of biological processes. Although one cannot conclusively claim the predictive superiority of the *WNC*-based method on the basis of these observations, this analysis provides additional evidence of the potential predictive sensitivity and specificity of this approach.

These comparisons suggest that, in principle, the *WNC* measure can find gene subsets that underlie biologically meaningful associations. However, a caveat in this type of analysis is the dependence on the availability of GO terms for the genes investigated. This may represent a critical constraint in relatively less well characterized genomes, such as the zebrafish genome. Because of the current interest in generating novel hypotheses about the regulatory mechanisms sustaining heart regeneration after injury in this organism, the following section offers a deeper view into the ZF network predictions.

### Application Case: Heart Regeneration In Zebrafish

Unlike mammals, the zebrafish displays a remarkable ability to regenerate heart tissue after substantial damage. Because of this and other clinically interesting attributes, this organism has become a promising model to understand adult myocardial regeneration *in vivo*[42-44]. Moreover, the aim is to translate such knowledge into novel therapeutic strategies that can boost this heart healing property in humans. To tackle this challenge, a combination of experimental and computational approaches to identifying the biological regulatory networks responsible for driving cardiomyocyte proliferation is needed.

**Figure 5:**
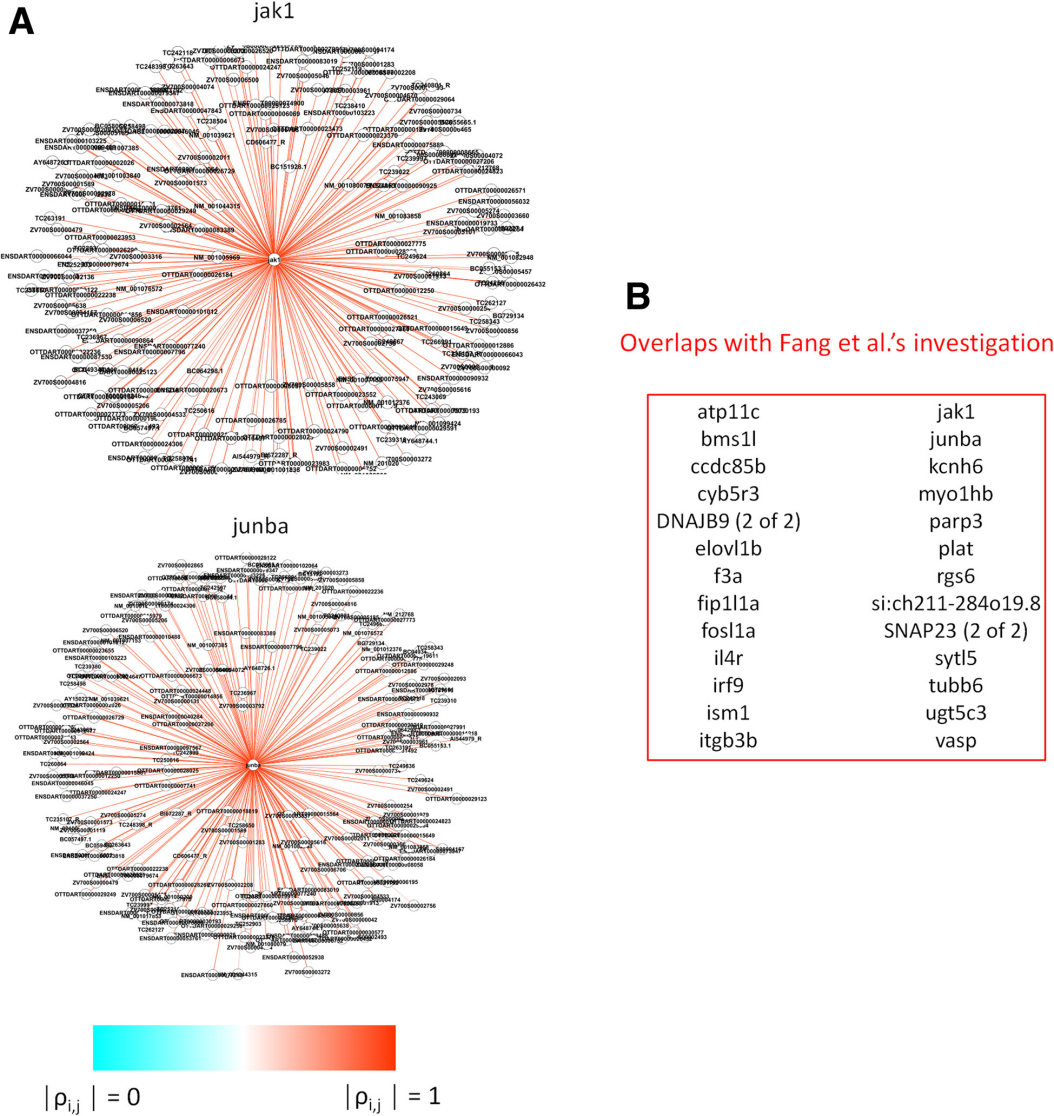
**Top-ranked genes detected by *WNC *score analysis. A**. Examples of top predictions. **B**. Top genes (*WNC* scores with *P* < 0.05) that were also found as biologically relevant components of heart regeneration in a recent study by Fang et al. [28]. Their approach was based on gene differential expression analysis and independent experimental validations.

Here, I had a closer look at the genes detected as potentially interesting by the *WNC*-based approach (Dataset S3). These genes are not only statistically linked to the regulation of immune responses (previous subsection), but also are known to be implicated in cell proliferation and differentiation. Genes with highly influential roles in adult zebrafish heart regeneration, such as jak1 and junba, were identified with high statistical confidence (Figure 5A). Fang et al. [28] recently demonstrated that Jak1/Stat3 pathway is a major regulator of cell proliferation and regeneration after injury. Fang et al. showed the regulatory capacity of jak1 based on standard differential expression analysis followed by stringent experimental validations [28].

The findings from Fang et al.’s study overlap with the predictions based on top-ranked *WNC* scores: 26 genes in total (Figure 5B). Furthermore, the *WNC*-based method highlighted the potential driving roles of other genes in tissue regeneration, such as those involved in upstream signaling (tnfrsf11b) and the Wnt signaling network (rhoab). Although these findings merit further investigation, they show the capability of *WNC* scores to recognize known drivers of cardiac regeneration and possible novel candidates.

To further illustrate the differences between the *WNC* scores obtained from the input network and those obtained from the randomized networks, a GO enrichment analysis of top-ranked genes from 5 different randomized networks were performed. The selection of top-ranked genes from the randomized networks was based solely on the *WNC* scores obtained. The *WNC* scores from these networks are plotted in Figure 1. This analysis focused on 108 genes, which is also the number of genes identified in the input (real) network. None of the selected gene sets from the random networks reported statistically significant (*P* < 0.05) enrichments of biological processes (DAVID tool).

**Figure 6:**
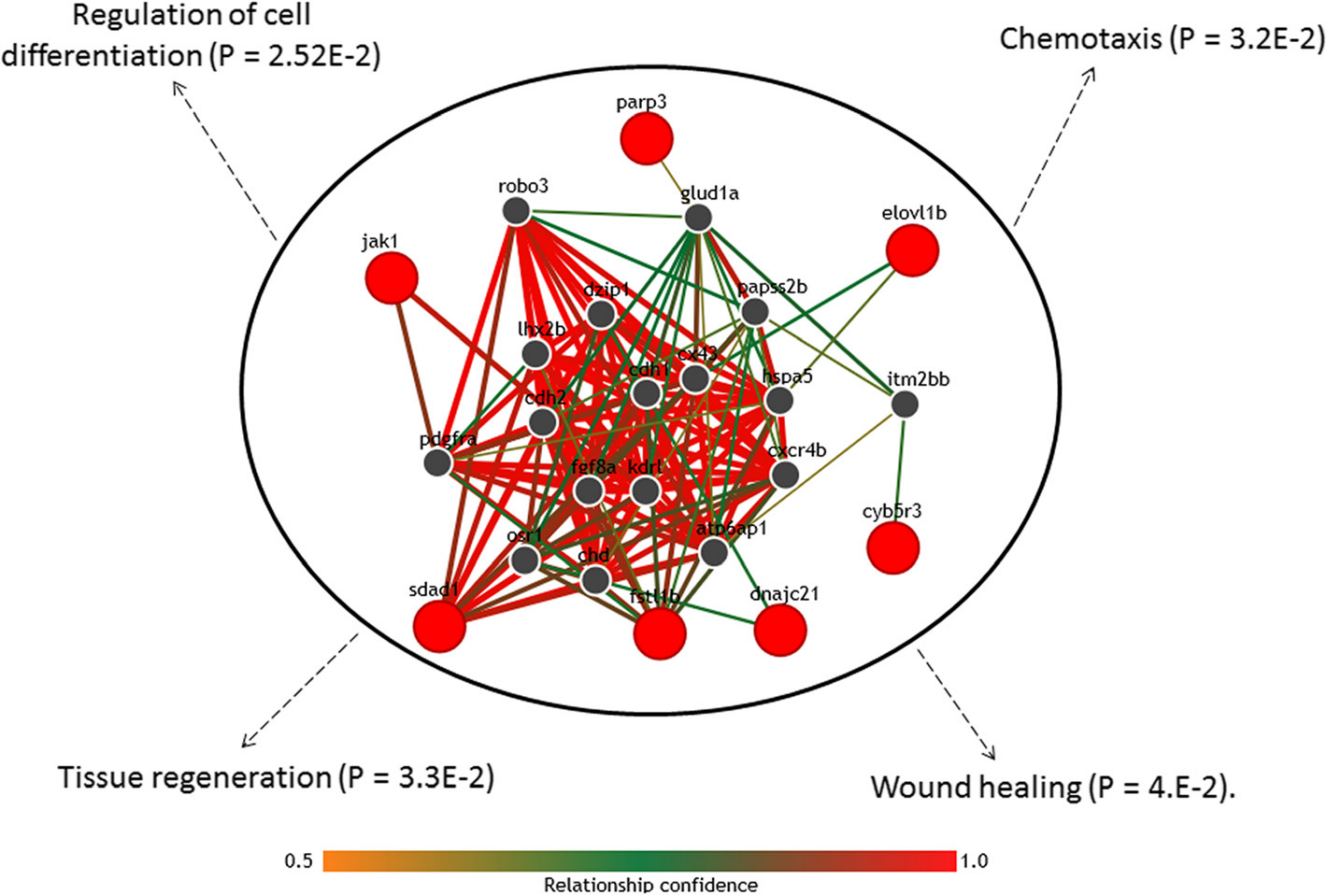
**Computational prediction of associations between the top candidates in the ZF network and diverse biological processes.** High-confidence associations with genes and cellular processes obtained from the IMP analysis. In the network, red nodes represent genes predicted as top candidates in the ZF network using significant *WNC* scores. Grey nodes represent other genes predicted by IMP to be functionally associated with the top ZF network genes. Color bar indicates the level of statistical confidence of the predicted gene-gene associations. Examples of biological processes significantly enriched in this predicted network are indicated.

To further investigate the potential relevance of the top-ranked genes derived from the *WNC*-based method, functional associations between these genes and biological processes were computationally predicted with the IMP system (Methods). IMP found 45 biological processes substantially associated with the *WNC*-detected genes (*P* < 0.05). These associations range from the “regulation of anatomical structure morphogenesis” (*P* = 6.9E-4) and “regulation of cell differentiation” (*P* = 2.52E-2), to “tissue regeneration” (*P* = 3.3E-2) and “wound healing” (*P* = 4.E-2). Figure 6 illustrates examples of statistically reliable predictions generated by this analysis. It also includes genes inferred by IMP to be functionally associated with the *WNC*-predicted genes in the ZF network.

### Alternative methods for edge weight aggregation

To offer initial insights into suitable alternative approaches to aggregating edge weights, other two scores, *WNCB* and *WNCC*, were implemented as follows (notation as defined above):

(6) $$\mathrm{WNC}B_{i}=\;\frac{\sum_{j}^{N}w_{i,j}}{N}$$

representing the mean of the observed edge weights.

(7) $$\mathrm{WNC}C_{i}=\;\sum_{j}^{N}{w_{i,j}}^{2}$$

corresponding to the sum of squared weights.

The motivation for its introduction here is to offer connectivity scores in alternative scales, including a normalized version of the score in the case of *WNCB*. In principle, they are suitable alternatives because they are based on the original *WNC* implementation. They have not been yet investigated in the specific context of gene co-expression networks.

A comparison of *WNC*, *WNCB* and *WNCC* indicates that their estimated statistical significance and rankings are highly consistent. For instance, in the case of the ZF network, the *P* values obtained from the *WNC*-based method are significantly linearly correlated with *WNCB* (Spearman coefficient, ρ = 0.98 with *P* = 2E-7) and with *WNCCC* (ρ = 0.84 with *P* = 2E-7). These results show that these edge aggregation methods provide the basis for concordant gene rankings. Although, in principle, this should be expected in relation to *WNCB*, further characterizations of the potential predictive power and advantages of these and other methods deserve to be investigated independently as part of future work.

### Software availability

An executable program (WiPer), user instructions, source code and sample files can be downloaded, under the terms of the General Public License, at: http://sourceforge.net/projects/wipersoftware. The only user-defined inputs that the tool requires are: the co-expression network, a list of genes in the network to be analyzed and the number of permutations to estimate statistical significance.

## Discussion

Gene centrality scores of co-expression networks offer complementary approaches to extracting biological knowledge. They are useful to select genes with significant connectivity patterns, which may be biologically meaningful. This is done under the premise that genes with many, strong network connections are: a. likely to be co-regulated with their co-expressed genes, and b. influential in phenotype-specific cellular processes.

Here I investigated the *WNC* score, a measure of gene centrality for co-expression networks. *WNC* allows the automatic selection and ranking of genes based on a weighted version of node degree, together with a probabilistic assessment of its significance. This study has shown that, in relation to random networks, real phenotype-specific co-expression networks generate larger *WNC* values. This method was compared to 4 standard measures of centrality, which can also be applied to select candidate targets, such as candidate hubs and high-traffic nodes. Using three independent gene co-expression networks from different application domains, the *WNC*-based method generated gene sets that tend to be more enriched in cellular processes in comparison to a selection of genes highlighted by standard scores.

The proposed method allows the ranking and selection of candidate hubs based on an assessment of the statistical significance of their connectivity scores. This is done here with a randomized, permutation-based test. The problem of generating meaningful null models in weighted networks is still an open problem. Also previous research [45] have highlighted the limitations of large-scale correlation screenings that rely on the definition of correlation thresholds. The critical problem is that for datasets with more variables (genes) than samples, a relatively high number of false discoveries (false relevant correlations) can be detected even in the case of very high correlation thresholds. The method proposed here does not define or require pre-defined correlation thresholds.

Genes exhibiting statistically significant *WNC* values are consistent with prior knowledge. This was observed in two co-expression network models. The *WNC*-based analysis found groups of genes involved in metabolism and cell growth in the case of the GBM network. In the second case, top-ranked predicted genes were strongly associated with cell proliferation, differentiation and tissue repair following heart injury in zebrafish. Similarly, the method provided gene sets that overlap with recent research that involved rigorous experimental validations.

These findings suggest that *WNC*-based gene centrality analysis may help researchers to discover novel and interesting genes. It represents a tool for exploratory analyses and for making sense of complex co-expression networks. Further investigations, including additional computational comparisons and applications, are warranted. The method is currently being applied to other domain-specific projects followed by independent experimental validations. An easy-to-use, platform-independent software implementation of the method is provided to enable similar efforts elsewhere.

Currently we cannot conclude on the robustness of the proposed technique against variations in different application-driven design conditions, including: choice of co-expression measure, co-expression threshold definition for unweighted network methods, selection of method for multiple-testing corrections of the *WNC*-derived *P* values, and the definition of gene prioritization schemes for standard centrality scores. Furthermore, the robustness of the different techniques available to each choice category in different applications has not been conclusively demonstrated elsewhere. As these issues can be mapped to a myriad of data-specific and application-dependent conditions, the crucial question may not be whether the proposed method is robust against all these choices. But instead: Under which specific conditions, for each design choice, the method may be robust? An answer to this question will require their own comprehensive investigations.

Regarding choice of co-expression measures, one should expect differences in network generation and gene prioritization outcomes depending on the characteristics of the data. A wide diversity of measures are available, and previous research has shown the pros and cons of each option in different applications [6,46,47]. Deeper investigations of the effects of measure choice on the structure and function of co-expression networks is necessary. Regarding co-expression threshold definition for unweighted network methods, Perkins and Langton [11] highlighted the complexities associated with this task. Their investigation made the case for conservative threshold selection approaches, including one that is shown as more stringent than the selection based on the top 1% of co-expression values [11]. This challenge, which is also unlikely to lead to standard solutions, will continue to be a subject of additional investigations. Regarding the selection of method for multiple-testing corrections of the *WNC*-derived *P* values, one should expect that different techniques will report different numbers of statistically detectable *WNC* values. Here I focused on the Bonferroni correction not only because of its ease of implementation, but also because it offers conservative estimates of statistical significance. At least as a starting point in exploratory research, the challenge of reducing potential false positives is a more pressing concern than improving prediction power. Future versions of the WiPer tool will incorporate alternative techniques for multiple-testing correction. Lastly, the problem of defining approaches to selecting candidate genes from standard centrality scores will require further investigations. Here I showed that a conservative selection of top-ranked genes (those above the 0.95-percentile) and one that includes the same number of genes detected by the *WNC* score are restricted in terms of their functional enrichment detection potential. Despite these findings, there is a need for additional independent comparisons. To aid users in developing new applications and to facilitate other investigations that address these challenges, a free and easy-to-use implementation of the proposed method is available.

The analysis presented here focused on networks in which between-gene co-expression was calculated with the Pearson correlation coefficient. Although the proposed method does not constrain the user in the selection of the gene-gene similarity measure, it would be useful to further investigate its application to different types of networks that are defined by diverse measures, including non-linear association techniques. This is important because there is no standard way to generate these networks, and because the selection of gene similarity approach is a problem-specific task [6].

## Conclusions

This investigation makes three main contributions to the analysis of co-expression networks. First, a statistical indicator of gene centrality based on the size and strength of co-expression connections was developed. Second, an algorithm that selects statistically relevant genes was implemented and compared with standard centrality scores. This study shows that the proposed method complements and expands the predictive capacity of other techniques. And lastly, the potential of the proposed algorithm to generate biologically meaningful insights was illustrated in different application areas.

## Reviewers’ comments

I thank the reviewers: A. Almudevar, D.P. Kreil, M.M Kandula (working with D.P. Kreil) and C. Wells for their constructive and thorough evaluations. A point-by-point response to their comments follows. A full response, including those comments indicated as “not for publication” by the reviewers, were provided via the manuscript submission system.

### Reviewer 1 (Dr A. Almudevar) - Round 1

“The paper is interesting, well written, and it addresses an important open problem. The main concern I have is with the definition of the null model, discussed largely on page 6. Is there anyway to evaluate whether or not whether the null model used is appropriate, possibly by using simulated models? An inappropriate null model can lead to spurious reports of significant connectivity.”

Major points:

“1. On page 6: “The latter was estimated by randomly shuffling the edges in the network, while preserving the number of observed edges for each gene.” This constraint can be sufficient to completely specify the graph (for example, for N nodes, specifying N-1 edges for node 1 and one edge for the remaining nodes).”

**Response:** I realize that “shuffling” may not be the most accurate term to describe the procedure.

A more suitable description is “swapping”, because for each gene pair in the network its edge (weight) is randomly swapped with the edge (weight) from another gene pair in the network.

This guarantees not only that the global network size and degree distribution are preserved, but also it ensures that only viable connections (and their corresponding weights) are used to build the null distribution. Thus, the resulting null distribution is both random and feasible.

“2. No justification for the randomization procedure is given, other than its use in cited papers [1,14-16]. Is it possible to say more about the appropriateness of this null model? My understanding is that this is very much still an open problem. See, for example, Hero and Rajaratnam (2011) [45] “Large scale correlation screening” JASA.”

**Response:** In the Section “Weighted node connectivity”, the justification is now explicitly stated as:

This permutation procedure is suitable to generate a null model because the resulting networks are random and comparable with the observed (original) network. In particular, this procedure guarantees the preservation of fundamental properties of the original network: size, degree distribution and weight distribution [18].”

Additionally, I agree with the reviewer that the problem of generating meaningful null models in weighted networks is still an open problem. Also I appreciate the recommended reference by Hero and Rajaratnam [45]. Hero and Rajaratnam’s article highlights the limitations of large-scale correlation screenings that rely on the definition of correlation thresholds. The critical problem is that for datasets with more variables (genes) than samples, a relative high number of false discoveries (false relevant correlations) can be detected even in the case of very high correlation thresholds. The method proposed in my article does not define or require pre-defined correlation thresholds.

I’ve added this comment in the Discussions.

“3. Does the expression in equation (3) vanish if the graph is not connected?”

**Response:** As the reviewer pointed out, equation (3) will only return values for nodes that are connected to other nodes. Also the closeness centrality value of a node is calculated in relation to the connected graph in which the node is located. This is a key limitation of this approach, which also implies that nodes located in small sub-networks (separated from the largest connected sub-network) may report relatively high closeness centrality values. In this investigation, all unweighted networks analyzed with this score consisted of a large connected graph containing the vast majority of the nodes, and all of the nodes had at least 1 edge and reported closeness centrality values between 0 and 1.

This observation is included in the revised version.

Minor corrections

“Page 5, line -3: “[w]here node …””

Corrected.

“Page 8, line -2 “betweness””

Corrected.

“Page 9, Left side of equation 5 depends on “j,k”. Is this correct?”

Yes, but in the sense that all neighbors of *i*, i.e., nodes *j* and *k*, are required for calculating the score. I have clarified it as follows.

$$u_{i}=\;\frac{m_{i}\left(j,k\right)}{m_{i}}$$

Where *m*_*i*_(*j,k*) is the number of edges connecting all nodes *j* and *k* in the neighborhood of node *i*, and *m*_i_ is the total number of edges that could be seen if all the nodes in the neighborhood of *i* were fully connected.

### Reviewer 1 (Dr A. Almudevar) - Round 2

Additional comments were not received.

### Reviewer 2 (Dr. D.P. Kreil) – Round 1

“The manuscript by Francisco J Azuaje investigates the significance of the connections in undirected gene network graphs as predicted from gene coexpression. Empirical pvalues are constructed for a sumscore (‘Weighted Node Connectivity’) by shuffling edge weights (coexpression measures). They are then used to select genes which are presumably biologically informative. Assessments of significance in such correlation graphs are certainly topical and of interest to the field. The presented work, however, raises a number of questions. ”

“For instance, in the section on ‘Weighted node connectivity’ the author first states that an assessment of statistical significance is needed for ranking genes. He then observes that ranking by the introduced empirical pvalues is equivalent to ranking by Weighted Node Connectivity alone (except for ties that arise from the limited number of shuffled samples). That suggests that the structure of the network graph does not affect the significance of a particular edge, and that the main purpose of the exercise seems to be the determination of a more principled cutoff for the edges to examine next. Threshold selection for gene coexpression analysis is a nontrivial task, and this should be emphasized in the manuscript and prior work should be referenced (see, e. g., Perkins & Langston, 2009 [11]).”

**Response:** My expression “the genes could be ranked by using either the WNC scores or their corresponding P-values” led the reviewer to this interpretation: “ranking by the introduced empirical *P* values is equivalent to ranking by Weighted node connectivity”. My explanation was not clear enough. A more accurate, clearer statement is:

In principle, the user could rank the genes by using either the WNC scores or their corresponding P values. This is because the larger the WNC score, the more likely that the gene represents a candidate hub. However, a ranking based on only P values should be used because it provides a more reliable estimator of gene connectivity. Indeed, it is possible to obtain genes with relatively high WNC scores that are detected as statistically spurious after P value calculation. This is reflected in the observation that WNC scores and their corresponding P values are not perfectly linearly correlated (Additional files 1, 2 and 3). Pearson correlations range between -0.53 (for the ZF network) to -0.85 (for the GBM network). Moreover, P values represent more interpretable values to bioinformaticians and experimental biologists alike, e.g., values between 0 and 1 with well-defined statistical meaning.

The section on ‘Weighted node connectivity’ now includes this revision. Also, in the Introduction, now I refer to Perkins & Langston (2009), as recommended.

Perkins AD, Langston MA. Threshold selection in gene co-expression networks using spectral graph theory techniques. BMC Bioinformatics. 2009;10 Suppl 11:S4.

“Critically, however, the actual improvements over established approaches that could be achieved by the proposed method are not sufficiently clear. The sensitivity of the method is assessed by GeneOntology enrichment analysis. There is no assessment of specificity though. In particular, the question arises if the observed differences in GeneOntology results are not entirely or, at least, largely caused by differences in the numbers of genes selected. The results presented in the section “Comparison with standard gene centrality methods” actually indicate that this may well be the case, as a reduction in the number of genes obtained by applying stricter thresholds in the presented method yields worse results. Benchmarks should thus directly assess performance as a function of the number of genes selected, parameterized by threshold stringency. Comparisons of different methods of network construction, including the various unweighted centrality measures discussed, thus need to be parameterized to yield similar numbers of genes, to avoid confounding effects of gene set size and network construction. In particular, with the results from the proposed method with more genes selected apparently giving better GeneOntology enrichment, these comparisons should be made for methods parameterized to yield this higher number of genes. It is not meaningful to just show that the new and the established methods perform badly when selecting too few genes.

The current work does not provide these separate analyses, which are required to support the main claims of the paper.”

**Response:** To address this comment, I performed an independent GO enrichment analysis of larger sets of genes that were top-ranked by the standard methods. For each network and standard measure, the number of genes analyzed was equal to the number of genes detected by the WNC score. The results of this analysis are now included in the revised article, as follows:

To investigate the possibility that the perceived advantages of the *WNC*-based predictions over standard techniques is explained by the relatively larger gene sets detected by the former method, an independent GO enrichment analysis that accounts for this difference was implemented. The statistical enrichment of GO biological processes of top-ranked genes from each technique was estimated (DAVID tool, statistical significance at *P* = 0.05), in which the numbers of genes analyzed were equal to those detected by the *WNC* score: 55 genes (GBM network), 79 genes (KL network) and 108 genes (ZF network). In the GBM network, only the clustering coefficient predicted genes significantly enriched in biological processes (3 in total), which is consistent with the results previously obtained. In the KL network, the betweenness centrality score preserved its capacity to detect significant enrichments (17 in total). This time the genes identified by the clustering coefficient did not report significant associations. The degree-based method improved its predictive potential (14 significant enrichments), which is still below the number of statistically detectable associations that the *WNC* score reported. In the case of the ZF network, none of the standard methods reported significant enrichments of biological processes. Although one cannot conclusively claim the predictive superiority of the *WNC*-based method on the basis of these observations, this analysis provides additional evidence of the potential predictive sensitivity and specificity of this approach.

“Finally, although a framework is suggested for a more principled selection of the number of edges/genes, it is unclear how robust this number is in relation to a multitude of ad hoc design choices of the presented empirical algorithm:

i. Choice of coexpression measure. This can affect results: See, e.g., the discussion in Simon & Tibshirani (2011) [46] who recommend the distance correlation measure proposed by Székely & Rizzo (2009).

ii. Arbitrary threshold used for selecting a graph edge (correlation > 95%) in the unweighted methods. Although the author mentions trying alternatives above the 75% ile, results are not shown, and prior work on threshold selection has not been taken into account (e. g., Perkins & Langston, 2009).

iii. Bonferroni correction method for multiple testing, 5% FWER threshold.

iv. 95% ile threshold of centrality measures for comparisons of other methods.

Moreover, arbitrary thresholds are used for selecting genes from the examined data sets, and the procedure seems to be different for each data set, with little motivation or justification, nor is the robustness of results towards these choices examined.”

**Response:** First of all, I agree with the reviewer that these 4 factors are critical in the evaluation of this and any related approach. At the same time, I’d like to stress that I am not (cannot) claim that my method is robust to all these choices and the variety of options that are in principle available to each choice. Furthermore, the robustness of the different techniques available to each choice category for different applications has not been conclusively demonstrated elsewhere either. Also I think that the crucial question may not be whether my method is robust against all these choices. But instead: under which specific conditions, for each design choice, the method may be robust? An answer to this question will require their own comprehensive investigations. Also note that a more detailed response to Point iv. was provided above.

In the Discussion, now I address this concern, including a point-by-point comment on the 4 choices, as follows:

Currently we cannot conclude on the robustness of the proposed technique against variations in different application-driven design conditions, including: choice of co-expression measure, co-expression threshold definition for unweighted network methods, selection of method for multiple-testing corrections of the *WNC*-derived *P* values, and the definition of gene prioritization schemes for standard centrality scores. Furthermore, the robustness of the different techniques available to each choice category in different applications has not been conclusively demonstrated. As these issues can be mapped to a myriad of data-specific and application-dependent conditions, the crucial question may not be whether the proposed method is robust against all these choices. But instead: Under which specific conditions, for each design choice, the method may be robust? An answer to this question will require their own comprehensive investigations.

Regarding choice of co-expression measures, one should expect differences in network generation and gene prioritization outcomes depending on the characteristics of the data. A wide diversity of measures are available, and previous research has shown the pros and cons of each option in different applications [6,46,47]. Deeper investigations of the effects of measure choice on the structure and function of co-expression networks is warranted. Regarding co-expression threshold definition for unweighted network methods, Perkins and Langton [11] highlighted the complexities associated with this task. Their investigation made the case for conservative threshold selection approaches, including one that is shown as more stringent than the selection based on the top 1% of co-expression values [11]. This challenge, which is also unlikely to lead to standard solutions, will continue to be a subject of additional investigations. Regarding the selection of method for multiple-testing corrections of the *WNC*-derived *P* values, one should expect that different techniques will report different numbers of statistically detectable *WNC* values. Here I focused on the Bonferroni correction not only because of its ease of implementation, but also because it offers conservative estimates of statistical significance. At least as a starting point in exploratory research, the challenge of reducing potential false positives is a more pressing concern than improving prediction power. Future versions of the WiPer tool will incorporate alternative techniques for multiple-testing correction. Lastly, the problem of defining approaches to selecting candidate genes from standard centrality scores will require further investigations. Here I showed that a conservative selection of top-ranked genes (those above the 0.95-percentile) and one that includes the same number of genes detected by the *WNC* score are restricted in terms of their functional enrichment detection potential. Despite these findings, there is a need for additional independent comparisons. To aid users in developing new applications and to facilitate other investigations that address these challenges, a free and easy-to-use implementation of the proposed method is available.

“In order to convincingly show the value of the presented approach, the author needs to address the above questions, especially regarding a separation of the effects of gene set size on the downstream GeneOntology analysis used to gauge performance.”

**Response:** A detailed response, including new results, regarding gene set size effects on the GO analysis is provided above.

### Reviewer 2 (M.M. Kandula, working with Dr. D.P. Kreil) – Round 2

“We appreciate the correction of the original statement claiming that the P-values and WNC scores were linearly correlated, when there are clear differences as reflected in correlation coefficients as weak as -53%. Consequently, the revised text seems misleading: It is then not irrelevant whether genes are ranked by WNC scores or P-values. On the contrary, the permutation procedure suggested by the author to compute these P-values seems to be a key step in the proposed algorithm, and we think that this should be made clear in the manuscript. Alternatively, if it really made no difference then why would the author introduce that elaborate step?”

**Response:** This point is now clarified in the article as follows: “Ranking and selection of genes should be based on their *P* values. This is because, in comparison with *WNC* scores, *P* values provide a more reliable estimator of gene connectivity.”

“We appreciate the additional GO enrichment analysis of larger, size-matched sets –supporting the claims made. We maintain that the comparisons with deviating set sizes were not meaningful and are actually misleading by giving the wrong impression that there were additional independent evidence. Those results from unmatched data sets should thus be removed from the presentation.”

**Response:** Although I agree with the reviewers that the results from unmatched datasets do not offer sufficient evidence, I believe that the findings from both size-matched and –unmatched sets are relevant in this publication. To further address the reviewers’ concern, I have added the following statement (following results from unmatched sets): “Although the selection of gene sets from the traditional methods is viable in a typical network analysis, it is necessary to caution that a fairer comparison of performance with the proposed method should be based on size-matched datasets.”

“While we find it disappointing that the author has made no attempt to test for robustness of the proposed new approach by examining the stability of results under varying algorithm choices and parameters, the revised manuscript now includes an appropriate disclaimer.”

“While we appreciate the extensive disclaimer added, we respectfully disagree with the view that an assessment of robustness of results were out of scope: At least initial attempts at establishing that a new approach does not only work for a particular arbitrary set of algorithm choices and parameters would give other scientists some evidence that the illustrated performance improvements should apply more generally and are really due to the presented new ideas.”

“While we appreciate that the source code has been made available we find it disappointing that not even some basic investigations into the robustness of the presented approach are presented by the author.”

**Response:** Although I agree with the reviewer that an analysis of the robustness of the algorithm against different user-defined input choices is relevant, it is also important to clarify that the reviewers’ concern mainly refer to user choices and parameters that are not defined by the proposed algorithm per se. Rather, they refer to conditions related to input data selection.

[Regarding the previous author’s response on gene set size effects on the GO analysis, the reviewer states:] “Thank you for the new analysis supporting the claims”.

“If the author does not wish to demonstrate robustness under threshold selection then the used thresholds added should at least be motivated/justified. It is entirely unclear why, for onedata set, ‘|log2 fold change|’ ≥ 1 was used for calling ‘significant expression change’ while, for another, an ‘adjusted P-value = 0’ and, for yet another, an ‘adjusted P-value = 0.05.’”

**Response:** In the GBM dataset, log_2_Fold-Change was used to select genes, instead of *P* values, because the latter detected relatively low numbers of differentially expressed genes. In the other datasets, the chosen *P* values allowed the selection of gene sets consisting of hundreds of genes instead of thousands. In all datasets, the latter was the main selection requirement. This clarification is now included in the manuscript.

“Moreover, these statements are imprecise: One can either apply a P-value threshold = some value or select genes with P-value < some threshold. It is moreover not clear what the author means with ‘adjusted P-value = 0’ - perhaps a typing error?”

**Response:** Corrections made: *P* < threshold (In KL dataset). In the ZF dataset: only those genes with adjusted *P* = 0 were selected. “Adjusted *P*” refers to *P* values obtained after correcting (adjusting) for multiple testing. The latter is also clarified in Methods.

### Reviewer 3 (Dr C. Wells) – Round 1

“Building informative biological networks from systems-scale datasets is an area of increasing importance, as data becomes more accessible, and more complex. There is a clear need to reduce complex networks to the most essential and informative core. The author has identified the connectivity of nodes in a network as a common method to highlight biologically relevant areas. The assumption, quite reasonably, is that ‘important’ network elements will be highly connected, thereby allowing researchers to focus on elements also likely to be highly functionally related.

I liked the concept of being able to select biologically relevant genes in a network very much, and I liked the idea that the ‘strength’ of a correlative score could be further filtered with some quantitative, reproducible and comparative metric. However I don’t think that the author has adequately addressed this goal in the manuscript in its current form.

An over-arching criticism that I have of the manuscript is the tendency to superficial generalization by the author. The introduction lacked depth of critical analysis on the problem at hand, rather suffering from too many soft introductions to generic network structures, and not enough specifics about the types of networks needing improvement (or why). Instead the author relies on generalizations like ‘coexpression networks’ of which there are many flavors. I would have appreciated a more detailed understanding of the author’s motivations, particularly with regard to the deficiencies in commonly used network approaches. The two main points made by the author ‘biological relevance’ and ‘binary thresholding’ remained undefined and essentially untested in this manuscript. I was intrigued by the idea that one might build a coexpression network without applying a threshold (and here I’m assuming the binary reference refers to Steve Horvath’s original weighted gene co-expression network model, which is by definition binary).”

**Response:** The main rationale is that traditionally users detect candidate hubs by counting the number of edges associated with a node. In the context of gene correlation networks, a connection is typically defined if the correlation between a pair of genes is above a predefined cut-off value. Here, I aimed to provide an approach to detecting candidate hubs without the need to pre-specify a correlation threshold to define edges in the network. Also there is a need to offer an automated way to quantify (and rank) the resulting candidate genes according to the statistical significance of the observed connectivity.

To address the reviewer’s concern, I have clarified my motivation in the Introduction.

“What does the author mean by “Moreover, the predictions of gene centrality generated by these measures are not commonly supported by probabilistic assessment at the level of individual genes, which is crucial for gene selection and prioritization”? In the first instance, this statement needs context to be understood and in the second instance I’m not sure it can be correctly stated, certainly not in this blanket way – there are many ways to build a network and few rely on a single measure of co-expression between network elements.

**Response:** I agree that this sentence does not clearly represent the intended message. Here I do not refer to the construction of the co-expression network or the estimation of between-gene correlations. I refer to a key current limitation: the idea of defining candidates hubs by either counting the number of edges assigned to a node or by estimating the total intensity of the connections without providing an indicator of statistical significance (P value) for each candidate hub.

To address this point, I’ve modified this sentence accordingly.

“The method itself builds on a method originally described by reference [14] - Zhang and Horvath (Stat Appl Genet Mol Biol. 2005;4:Article17) which discretizes a fairly well established linear correlation (Pearson) correlation methods. It’s not clear how the other two references included here build the argument of the author, or demonstrate modifications to the method that the author is relying on. Furthermore there have been very strong arguments made by Steve Horvarth, amongst others, that improved correlation networks may also require nonlinear measures to assess connectedness between nodes. This makes the method feel somewhat redundant from the outset.”

**Response:** Please note that my method does not discretize or dichotomize the correlations between genes. The method is proposed to overcome this constraint.

I agree that meaningful co-expression networks may require non-linear measures of correlation. However, an investigation of the selection of suitable correlation measures for different biological network applications is beyond the scope of my study and has been addressed elsewhere, including by Horvarth et al. [6]. The method that I propose does not constrain the user in the selection of gene similarity measure. In this context, the user is responsible for providing the network to be analyzed.

I have made clarifications in the sections: Weighted node connectivity, Generation of gene co-expression networks, and Software Availability.

Also, in Discussions, I have stated that:

The analysis presented here focused on networks in which between-gene co-expression was calculated with the Pearson correlation coefficient. Although the proposed hub detection method does not constrain the user in the selection of the gene-gene similarity measure, it would be useful to further investigate its application to different types of networks that are defined by diverse measures, including non-linear association techniques. This is important because there is no standard way to generate these networks, and because the selection of gene similarity approach is a problem-specific task [6].

“I’m assuming that the absolute value of the Pearson correlation wi,j means that the network does not discriminate between positive or negative correlations. I’m not sure that the WNC would cope with negative values, but ignoring these means that valuable information about the type of relationship that an edge may represent is lost.”

**Response:** Yes, the absolute correlation values are used to estimate the connectivity scores (WNC scores). This is actually done to not exclude any type of correlation. This means that the user can identify candidate hubs defined by either strong positive correlations, strong negative correlations, or a combination of both. Once candidate hubs have been detected, the user can directly inspect in his/her data the type of correlations associated with each hub.

“The method next builds a distribution model of weighted network connectivity scores WNC and permutes a significance value by comparing a shuffled-edge network (of random values) with the original network. The motivation is to reduce edges between poorly correlated nodes. In essence, introducing a threshold. I need a better explanation about why this threshold is an improvement over an arbitrary correlation threshold. In point of fact, the supplementary tables demonstrate that very few genes pass the Bonferroni-corrected P-values, so one must ask whether this threshold is less appropriate than the traditional correlation value or discretized approach.”

**Response:** This statistical test allows users to automatically detect and rank candidate hubs. The traditional method based on defining cut-off correlation values does not address this problem directly. It simply offers a simplified way to represent the network, and it still requires the definition of criteria for identifying candidate hubs.

I agree that the Bonferroni-corrected *P* values are conservative, and that additional potentially interesting genes may be loss in this selection. However, I think that, at least in an exploratory analysis phase, a reduction of potential false positive predictions is preferable that an increase in predictive power. Also note that although in this paper I focused on results significant at (corrected) *P* = 0.05, the method allows the user to define her/his own statistical significance criteria. The output of the tool includes the connectivity scores, together with nominal and corrected *P* values, for all genes in the network.

To address the reviewer’s observation, in the Discussion I have included this clarification and stated the importance of considering alternative multiple-testing correction techniques.“I’m also confused that the motivation for the study was phrased in terms of improving ‘biological interpretation’ but the choice of ranking and thresholding network members was on a statistical basis (p-value) because this was supposedly more understandable to biologists and statisticians. I’m especially confused given that the ZF-network in Figure 1 appears to offer no improvement over the randomized background.”

**Response:** In the revised version, I’ve rephrased the “improving biological interpretation” statement as “to help researchers to focus their attention on genes that may be biologically informative”. Here this is the case because now the user has access to a ranking of genes on the basis of their connectivity in the network and corresponding statistical significance support.

I have modified Figure 1 to show the connectivity scores obtained from different random datasets. As this figure and the subsequent results demonstrate, there are indeed gene sets with (observed) connectivity scores that are significantly high and unlikely to have been obtained by chance (at *P* = 0.05).“The author then makes the statement that “WNC scores and their P-values are linearly correlated.” In fact the P-values appear to discretize the data into not significant (close to 1) or highly significant and few elements sit in between. I don’t share the author’s interpretation of figures 1 and 2. Please justify this statement.”

**Response:** Although not highly correlated in all networks, *WNC* scores and corrected *P* values are indeed linearly correlated. Their (Pearson) correlations range between -0.53 (for the ZF network) to -0.85 (for the GBM network).

I have made this clarification in the section on *Weighted node connectivity*. These data are also included as supplementary files.“Figure 3 allows the reader to visualize the number of connections of a gene with a ‘strong’ WNC score, and contrasts it to one with a large number of ‘weak’ connections. However an independent measure is needed to assess the relevance or validity of these ‘weak’ or ‘strong’ interactions. And given the emphasis on biological relevance, is there any indication that the genes connected with a high WCA are more informative, and the genes connected with a weak value more spurious? The geneset-enrichment analysis wasn’t particularly convincing, and little detail was provided on the Col6A3 network in comparison to the Aruka network, and this section was altogether too anecdotal in nature.”

**Response:** The proposed algorithm offers statistical indication of the differences between those genes with weak and strong interactions, i.e., the estimated *P* values.Regarding the examples shown in Figure 3, I have included a more detailed discussion as follows:

AURKA is a kinase known to be implicated in the regulation of cell cycle progression and has been associated with different tumor types and treatment responses [36,37]. COL6A3 is a collagen protein that has been linked to different inherited muscular disorders [38]. The AURKA network includes 109 associations with absolute Pearson correlations above 0.80, including 36 correlations above 0.90. They include genes involved in the regulation of apoptosis: BLOC1S2, CSTB, LGALS1, PRDX3 and RTN4, and in microtubule cytoskeleton organization: ZWINT and BLOC1S2 (David tool). This set of highly correlated genes also includes IDH1 (Isocitrate Dehydrogenase 1), which has been proposed as a potential therapeutic target in gliomas [39]. In contrast, the COL6A3 network includes 120 associations with absolute Pearson correlations below 0.30. This low-correlated set includes genes linked to a variety of cellular processes ranging from protein translation (such as KARS and COPS5), RNA processing (such as RBM3 and RPL14) to metabolism (such as DERA and ATP5F1).

“What does the author mean in regards to tissue specificity, in regards to the gene rankings. How was this assessed in terms of actual expression restriction (assessed, for example, against the BioGPS datasets).”

**Response:** I agree that this point requires clarification. I did not perform an analysis of tissue specificity of expression data per se. I only showed that there is no overlap between the set of candidate hubs from the brain and kidney networks. This only indicates that these predictions are particular to these two organ-specific networks. This clarification is now included in section “Identification of phenotype-related genes based on *WNC* analysis”.

“I’m not convinced that examining gene overlaps between different methods (Table 3) is a reasonable comparison, and surely the size of the dataset (dominated by the authors method) will have an impact on GSEA. You are comparing gene lists of 10–20 members with genelists of 55–100 members. It’s hardly surprising that you didn’t see GO term enrichments for the majority of these methods.”

**Response:** To assess the potential influence of this factor, I performed an independent GO enrichment analysis of larger sets of genes that were top-ranked by the standard methods. For each network and standard measure, the number of genes analyzed was equal to the number of genes detected by the *WNC*-based score. The results of this analysis are now included in the revised article, as follows:

To investigate the possibility that the perceived advantages of the *WNC*-based selection over standard techniques is explained by the relatively larger gene sets detected by the former method, an independent GO enrichment analysis that accounts for this difference was implemented. The statistical enrichment of GO biological processes of top-ranked genes from each technique was estimated (DAVID tool, FDR < 0.05), in which the number of genes analyzed was equal to that detected by the *WNC* score: 55 genes (GBM network), 79 genes (KL network) and 108 genes (ZF network). In the GBM network, only the clustering coefficient predicted genes significantly enriched in biological processes (3 in total), which is consistent with the results previously obtained. In the KL network, the betweeness centrality score preserved its capacity to detect significant enrichments (17 in total). This time the genes identified by the clustering coefficient did not report significant associations. The degree-based method improved its predictive potential (14 significant enrichments), which is still below the number of statistically detectable associations that the *WNC* score reported. In the case of the ZF network, none of the standard methods reported significant enrichments of biological processes. Although one cannot conclusively claim the predictive superiority of the *WNC*-based method on the basis of these observations, this analysis provides additional evidence of the potential predictive sensitivity and specificity of this approach.

“Likewise I find the ontology enrichments in the zebra-fish case study too anecdotal to be convincing. I would like to have seen a comparison with the randomized network, for example, to convince me that the biology associated with the top ranked genes were generally emergent with the method.”

**Response:** Please note that observed and randomized networks are compared when estimating the *P* values for each gene. To address the reviewer’s comment I have added the following to the section “Application case: Heart regeneration in zebrafish*”*.

To further illustrate the differences between the *WNC* scores obtained from the input network and those obtained from the randomized networks, a GO enrichment analysis of top-ranked genes from 5 different randomized networks were performed. The selection of top-ranked genes from the randomized networks was based solely on the *WNC* scores obtained. The *WNC* scores from these networks are plotted in Figure 1. This analysis focused on 108 genes, which is also the number of genes identified in the input (real) network. None of the selected gene sets from the random networks reported statistically significant enrichments of biological processes (DAVID tool at *P* = 0.05).

“Overall, the ideas put forward have merit, and could offer a valuable adjunct to more traditional weighted correlation measure networks. However the author hasn’t sufficiently benchmarked his method relative to existing approaches, including the original Horvath method. The anecdotal nature of the manuscript provides little hard evidence that the method offers improved biological context either.”

**Response:** I agree that Horwath’s Weighted Correlation Network Analysis (WGCNA) can be used to select candidate hubs. However, unlike the method proposed in my article, WGCNA does not explicitly address the statistical uncertainty of the observed scores, i.e., their statistical significance is not assessed. Because of this, my method can be seen as an extension of WGCNA’s hub identification method. Both methods estimate connectivity scores as the sum of edge weights, but the WGNA’s method does not estimate their corresponding *P* values. Also note that in the section “Comparison with standard gene centrality methods”, I presented a comparison between the gene selections reported by my method and those obtained from the standard score as implemented in WGCNA:

“To further illustrate the importance of statistical assessment in the *WNC*-based technique, a set of top genes ranked exclusively on the basis of their *WNC* values was selected from each network (Additional files 1, 2 and 3 for the GBM, KL and ZF networks respectively). Note that this method for selecting candidate nodes is equivalent to that available in the Weighted Correlation Network Analysis (WGCNA) method [41]. To select these genes, a cut-off number of top genes was defined. To make this comparable to the non-WNC-based methods, this number was equal to the maximum number of genes retrieved from the standard methods in the GBM, KL and ZF networks (Table 3). Statistically significant associations with GO biological processes (at P = 0.05) were not detected in any of these settings.”

I hope that the clarifications and new results included in this response can persuade the reviewer that, despite the limitations of the study and the need for further investigations, its conclusions are grounded in harder evidence than initially appreciated.

### Reviewer 3 (Dr C. Wells) – Round 2

“I appreciated the care that the author took in revising this manuscript, and agree that it is much improved in terms of clarity and caveat. I had no further major concerns regarding the motivation or implementation of the methods described in this paper. I had a very minor comment on the Figure legend for Figure 1, where “random” is spelt “radom”. I am satisfied that the method offers a useful addition to the toolkit of bioinformaticians wishing to evaluate and rank the connectivity of genes within a network.”

**Response:** Figure legends have been corrected.

## Abbreviations

WNC: Weighted node connectivity; GBM: glioblastoma treatment dataset; KL: Kidney versus liver tissue dataset; ZF: Zebrafish dataset; GO: Gene ontology; IMP: Integrative multi-species prediction.

## Competing interests

The author declares that he has no competing interests.

## Supplementary Material

Additional file 1 Results from GBM network. Click here for file

Additional file 2 Results from KL network. Click here for file

Additional file 3 Results from ZF network. Click here for file
